# Placenta Accreta Spectrum Disorder in a Patient with Six Previous Caesarean Deliveries: Step by Step Management

**DOI:** 10.1155/2021/2105248

**Published:** 2021-09-13

**Authors:** Gloria Calagna, Salvatore Polito, Francesco Labate, Rosa Anna Guiglia, Francesca De Maria, Chiara Bisso, Gaspare Cucinella, Giuseppe Calì

**Affiliations:** ^1^Obstetrics and Gynecology, “Villa Sofia-Cervello” Hospital, Palermo, Italy; ^2^University of Palermo, Palermo, Italy; ^3^Obstetrics and Gynecology, “Villa Sofia-Cervello” Hospital, University of Palermo, Palermo, Italy

## Abstract

The definition *placenta accreta spectrum disorders* (PAS) introduced by FIGO (International Federation of Gynaecology and Obstetrics) indicates an abnormal, pathological adherence or invasion of the placenta. The growing worldwide incidence of this pathological entity, and the possible serious correlated surgical risks, has caused a significant increase in attention among the scientific community. Previous caesarean delivery and presence of placenta previa are the main risk factors for the onset of PAS. Here, we present the intriguing case of a 39-year-old woman, at the 33rd week of gestation, with six previous caesarean sections and with a diagnosis of placenta previa accreta. At our referral center for PAS disorders, we successfully managed this difficult case with the help of a multidisciplinary skilled team.

## 1. Introduction

*Placenta accreta spectrum disorders* (PAS) is a heterogeneous group of abnormalities of placental adherence or invasion, for which previous caesarean sections and placenta previa represent main risk factors [1]. The American College of Obstetricians and Gynaecologists (ACOG) reported a PAS incidence ten times during the last fifty years, probably with an increase of the incidence of caesarean deliveries (CDs) [2, 3]. The occurrence of PAS is 1-3% in patients without a history of CD and increases to 24% in the case of one CD, to 40-50% in the case of three CDs, and finally to 67% in patients with six CDs [3, 4].

PAS are traditionally classified according to the “invasion depth” criterion, identifying three different conditions of increasing severity: placenta accreta, increta, and percreta [5]. However, this is a histopathological classification and thus only retrospective: it does not provide information on treatment plans or in vivo anatomical and vascular features.

More recently, “ultrasound (US) prenatal PAS staging” has been suggested and introduced in our clinical evaluation of patients, based on the presence of so-called *US invasion signs*, in order to stratify the prenatal risk and postsurgery outcome [6]. It should be mandatory to perform an in vivo analysis of PAS cases by prenatal imaging to establish the correct management and define particular surgical management.

Here, we describe the challenging management at our referral center of a unique PAS disorder in a patient with a history of six caesarean sections, focusing on focusing on the importance on the collaboration of a skilled multidisciplinary group.

## 2. Case Presentation

A 39-year-old woman, G7 P6, with 6 living children by caesarean delivery, was referred to our center at 33.1 weeks of gestation with a suspected PAS. She was Caucasian, 19 BMI, with normal vital signs, a personal history of appendectomy, and a more recent surgical treatment of pelvic endometriosis. Blood tests were normal, there was no clinical evidence of vaginal bleeding, and there were no US signs of urinary tract obstructions nor of hydronephrosis. Fetal growth was adequate.

**Figure 1:**
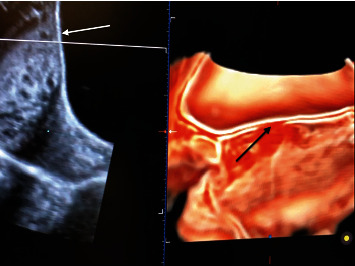
3D ultrasound image. White arrow indicates normal bladder line; black arrow indicates the perimetrial interruption.

**Figure 2:**
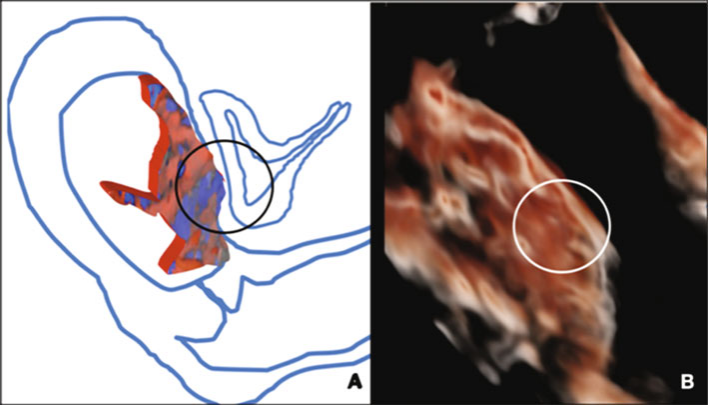
(a, b) Images of utero-bladder relationship. Graphic reconstruction (a) and 3D-ultrasound image (b) of the focal myometrial invasion without involvement of the bladder and integrity of the posterior bladder wall.

At presentation, ultrasound (US) examination showed an anterior placenta previa and a careful evaluation of prenatal PAS signs was performed by an expert sonographer (GC). Surprisingly, most of the PAS signs were absent: abnormal placental lacunae, bladder-line interruption, and myometrial thinning which was identified (Figure 1). The only ultrasound findings were the partial absence of the “clear zone,” that is, the loss of the hypoechoic plane in the myometrium underneath the placental bed and a moderate uterovesical hypervascularity (Figure 2). Diagnosis of PAS 1/2 was given [6].

After an in-depth study, implemented by a multidisciplinary team including gynaecologists, radiologists, neonatologist, and anaesthesiologists, it was decided to perform a CD at gestational age of 34 weeks, with a possibility of a hysterectomy, under support of highly qualified and experienced interventional radiology with temporary occlusion of hypogastric artery. After adequate counselling, the patient signed the informed consent form.

The patient underwent epidural anaesthesia. First, the interventional radiology procedure was performed, with a balloon catheter positioned in the hypogastric arteries, bilaterally. Subsequently, the caesarean section was performed according to the *Kustner technique*, on the previous scar [7]. Once the abdomen was opened, a large area dilated vessels were visible in the lower uterine segment; for this reason, we performed a vertical incision of the uterus in the upper part, away from the placental insertion, and a male foetus was extracted, alive and vital in breech presentation (Apgar score 9-10).

**Figure 3:**
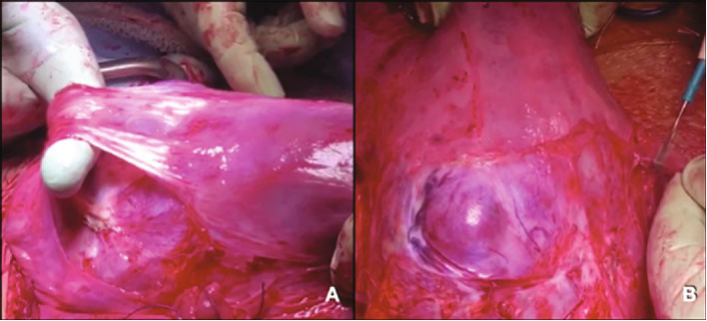
(a, b) Intraoperative images of vesicouterine peritoneum detachment (a) and evidence of placental “bulging” (b).

After clamping the umbilical cord and before carrying out the hysterectomy, inflation of the catheters previously inserted in the hypogastric arteries was performed. In the meantime, an inspection of the placental site was carried out: after developing a bladder flap clearing the lower uterine segment, the bulging of the invasion of the placenta in a very thin myometrium with intense vascularization was evident on the anterior surface of the external anterior uterus (Figure 3); no parameters and bladder infiltration were present. The uterine incision was closed leaving the placenta and umbilical cord stump in situ.

As a third surgical step, a total hysterectomy was performed, with ovary preservation, considering the patient's wish and her age. The uterus was submitted for histological examination, which confirmed the US diagnosis of placenta increta with focal percreta (PAS 1/2-US partial absence of the “clear zone” and a moderate uterovesical hypervascularity) [6].

During surgery, all vital signs were normal and stable. Intraoperative transfusion of one homologous blood transfusion was given considering an estimated blood loss of about 600 ml. On the 3rd postoperative day. she was discharged and the one-week follow-up revealed normal course.

## 3. Discussion

In the last decade, there has been a notable increase in incidence of CDs, with relative short- and long-term complications, such as slower recovery, rise in blood loss, infections, thrombosis, possible bladder, intestinal injury, and even hysterectomy [8]. The occurrence of PAS in successive pregnancies is a relevant risk, mainly due to the related maternal and fetal mortality and morbidity [8]. Despite the relevant clinical impact of the disease, PAS disorders remain undiagnosed before delivery in half to two-thirds of cases still today [9–11]. Given the difficulty and subjectivity in the interpretation of “typical” findings or signs by two-dimensional US and colour Doppler imaging; the recent literature reported variable sensitivity and specificity regarding markers of PAS [12]. However, there are no clear guidelines worldwide to define how and when to screen and evaluate women with previous CDs.

Recent studies have ascertained the histopathological correlation between caesarean scar pregnancy (CSP) and PAS disorders [13]. CSP is defined as the gestational sac located on the hysterotomy scar created by a previous caesarean section. This type of pregnancy is a pathological one, and if undiagnosed, it could have serious consequences and complications such as uncontrollable haemorrhage and need for hysterectomy [14]. Furthermore, an associated placenta previa in the setting of CSP suggests placental invasion. The diagnosis of CSP at the early first trimester is possible, and in this sense, CSP can be considered a surrogate marker for a future abnormally invasive placenta [15–18]. In particular, the study of the gestational-sac implantation site using COS (“cross-over” sign) criteria is a simple and reproducible tool for ascertaining the relationship between the ectopic sac, cesarean scar, and anterior uterine wall [19, 20].

Based on the possible severe consequences of a misdiagnosed PAS, patients may have the best possible outcome if referred to the closest “reference center” with multidisciplinary team for diagnosis, staging, and adequate management [21].

Our case demonstrates the importance of correct diagnosis and related surgical planning. The careful organization of the cesarean section at the 34^th^ week, the execution by highly skilled operators in election regimen (avoiding the urgency), and the support of intensive care unit and interventional radiology have allowed a desired and favorable result. In particular, reduced intraoperative bleeding was possible mainly thanks to the help of interventional radiology, achieving the reduction of the uterine vascularization during this delicate surgery and then ensuring a better maternal outcome.

In 2015, early first trimester US examination of pregnant women was incorporated into obstetrics and gynecology practice guidelines; it is a notable step forward in this field, because the identification of specific markers is a fundamental check point in order to carry out effective screening and therefore adequate diagnosis of a PAS disorder [22]. At the same time, the additional training in detecting the ultrasound signs of PAS using a standardized protocol has emerged as an essential tool significantly improving the diagnostic sensitivity of operators with only a basic obstetric ultrasound training [23]. In fact, high reproducibility and low interobserver variability of ultrasound imaging of PAS are essential to implement a screening program for women at a high risk of PAS [23].

Our experience highlights that a management by an experienced team reduces the risk of both maternal and fetal mortality and morbidity. In regional health facilities or smaller hospitals, which may lack the experience and the necessary support, PAS disorder (particularly the invasive forms) can result in numerous intra- and postoperative complications, such as massive haemorrhage secondary to attempts to separate the placenta from the uterus, in cases of pathology underdiagnosed. Based on the surgical risks and the perioperative challenges presented by PAS disorders, the transfer to a center of excellence relies on the recognition of the risk of PAS disorders, on accurate prenatal diagnosis, and on delivery with the help of a multidisciplinary experienced care team.

## Data Availability

Data are available on request (mail to pinocali13@gmail.com).
